# Effect of Anodization Temperature on the Morphology and Structure of Porous Alumina Formed in Selenic Acid Electrolyte

**DOI:** 10.3390/nano15241855

**Published:** 2025-12-11

**Authors:** Yulia V. Nazarkina, Vladimir B. Zaitsev, Daria A. Dronova, Alexey A. Dronov, Ilia I. Tsiniaikin, Danil D. Butmanov, Timofey P. Savchuk, Ekaterina V. Kytina, Elizaveta A. Konstantinova, Artem V. Marikutsa

**Affiliations:** 1 Institute of Advanced Materials and Technologies, National Research University of Electronic Technology—MIET, Bld. 1, Shokin Square, Zelenograd 124498, Russia; demetpatakai@gmail.com (D.A.D.); daa@miet.ru (A.A.D.); butmanovdanil@yandex.ru (D.D.B.); tamlon555@yandex.ru (T.P.S.); 2 Physics Department, M.V. Lomonosov Moscow State University, Leninskie Gory 1/2, Moscow 119991, Russia; ii.tcinyaykin@physics.msu.ru (I.I.T.); kata13012002@mail.ru (E.V.K.); liza35@mail.ru (E.A.K.); 3 Chemical Department, Shenzhen MSU-BIT University, International University Park Road 1, Shenzhen 518172, China; 4 Chemistry Department, M.V. Lomonosov Moscow State University, 1-3 Vorobyevy gory, Moscow 119234, Russia; artem.marikutsa@gmail.com

**Keywords:** porous anodic aluminum oxide, selenic acid, paramagnetic centers, F^+^ centers, photoluminescence

## Abstract

We report a comprehensive study on the effect of H_2_SeO_4_ electrolyte temperature on the composition, defect, morphological, and luminescent properties of porous anodic aluminum oxide (AAO). An increase in the synthesis temperature led to a decrease in the AAO cell diameter from 85–115 nm to 38–58 nm (depending on the electrolyte concentration) and enhanced the etching of the AAO walls, which even resulted in the disintegration of the AAO into individual fibers at 40 °C. The selenium concentration in the samples formed in 0.5–1.5 M H_2_SeO_4_ in the temperature range of 5–40 °C did not exceed 2 at.% and fell below the detection limit at 40 °C. The formation of a nanocrystalline Al_2_O_3_ phase was observed in the H_2_SeO_4_ electrolyte at 40 °C. The samples exhibited weak photoluminescence. We identified three types of paramagnetic centers in AAO formed in H_2_SeO_4_: F^+^ centers (N_sF_ = 8.2 × 10^15^ g^−1^), newly discovered centers with an unpaired electron localized on an oxygen atom (N_sO_ = 10^17^ g^−1^), and centers associated with selenate radicals (N_sS_ = 6 × 10^18^ g^−1^). By comparing the photoluminescence spectra and defect concentrations, we conclude that the luminescence of AAO formed in selenic acid is exclusively due to F^+^ centers, while other paramagnetic centers do not contribute.

## 1. Introduction

Porous anodic aluminum oxide (AAO) has become one of the widely used materials for nanostructure manufacturing, photonic crystals, optical and electrochemical sensors, drug delivery and biological nanocontainers, etc., due to its unique properties [1,2,3,4,5]. It is an inexpensive, chemically stable, and thermostable material with a high surface area. Its porous structure represents self-organized cylindrical pores, with a thickness proportional to the charge passed through the system and easily estimated, while the pore diameter is relatively constant over the entire sample area of the oxide layer. Meanwhile, the pore and cell diameters can easily vary from a few nanometers up to a micron by adjusting the technological parameters: the electrolyte composition determines the range of pore and cell diameters that can be obtained, and the anodization voltage or current density can be used to tune or modify these diameter directly during AAO formation. Moreover, by controlling both the oxide composition and morphology, it is possible to obtain either transparent or colored as well as either luminescent or almost non-luminescent AAO films [1,2,3,6].

Selenic acid is one of the promising electrolytes for AAO formation. Using this electrolyte, it is possible to rapidly produce highly ordered porous AAO both in potentiostatic [7] and galvanostatic [8] modes. An AAO structure with very low porosity and pore diameters of several nm can be obtained; meanwhile, the relatively large interpore distance allows for pore widening up to values close to 100 nm [9,10,11].

Despite the fact that porous aluminum oxide synthesized in selenic acid solutions possesses promising properties, there is still a lack of information about the influence of the technological parameters of the anodization process on the oxide. Although the influence of the electrolyte concentration, current density, voltage, anodization duration, and magnetic stirrer rotation rate on the morphology of AAO fabricated in selenic acid has been intensively studied [7,8,9,12,13], there is a lack of information about the effect of the anodization temperature. This is because the anodization temperature determines the electrolyte’s chemical activity and, as a consequence, the reaction rate, which eventually impacts the AAO’s composition and morphology, as has been shown for sulfuric and oxalic acids [14,15,16,17,18,19].

Although the majority of studies on aluminum anodization in selenic acid have concentrated on AAO morphology, only a limited number have investigated the influence of technological parameters on the composition and other properties of AAO. In particular, in [7], the composition of a single sample of AAO was studied using energy-dispersive X-ray spectroscopy (EDS). In [20], the same method was used to establish the influence of the oxide-formation voltage on its composition. In [11,21], the selenium content in Al_2_O_3_/NbO_2_ or Al_2_O_3_/ZrO_2_ composite samples, as well as in Nb or Zr rods doped with selenate, obtained by removing the AAO matrix, was studied in detail using X-ray photoelectron spectroscopy (XPS). Possible reactions occurring in the electrolyte and leading to selenium incorporation into composites were presented. However, studies of the composition of pure oxide structures without Nb or Zr have not been conducted. The calculated selenium concentration in porous structures varied significantly from article to article, ranging from 1% to 25% among different authors under similar AAO preparation conditions, indicating the need for a precise compositional analysis taking into account the specifics of each method.

Both the composition and morphology, depending on the anodizing temperature, ultimately affect its optical properties, such as reflectance and luminescence spectra, which are also crucial for the application of AAO in the above-mentioned fields [15,22,23,24]. AAO formed in 0.3–0.5 M selenic acid has been shown to be transparent [4,7] and does not exhibit parasitic effects in its Raman spectra even after annealing, as is the case for templates obtained with other electrolytes [8,25,26]. This feature makes AAO a promising material as a transparent, virtually non-luminescent matrix. However, there is still no consensus on which defects control the optical properties of AAO (especially luminescence), and whether the type and concentration of defects can be controlled by varying the synthesis conditions. Since the optical and electrical properties of porous alumina are largely related to paramagnetic vacancies and electrolyte inclusions [6,27,28,29], the most suitable method for studying AAO defects is electron paramagnetic resonance (EPR). EPR spectroscopy is a highly informative method for detecting point defects with unpaired electrons (paramagnetic centers), allowing one to determine the type of defects, their concentration, and local environment. Since defects with an unpaired electron actively interact with photons, such states can be responsible for photoluminescence. Thus, the EPR method is indispensable for establishing the nature of PL in AAO. To our knowledge, the identification of defects in AAO synthesized in selenic acid by EPR, the calculation of their concentrations, and their relationship with luminescence characteristics have not been studied in the international literature. Furthermore, we have not found any studies determining defect concentrations in AAO synthesized in any other acids. This prevents us from drawing definitive conclusions about the nature of the luminescence, as luminescence is negligible at low defect concentrations, while nonradiative recombination at defects may become dominant at excessively high concentrations. However, such information is essential for creating AAO -based optical sensors with specified characteristics. This paper presents a comprehensive study of the effect of H_2_SeO_4_ electrolyte temperature on the morphology and chemical structure of AAO, including defects. A combination of luminescence and electron paramagnetic resonance spectroscopy was used to determine the nature of defects in AAO and their concentrations.

## 2. Materials and Methods

### 2.1. Synthesis

Aluminum sheets with a thickness of 0.3 mm and a purity of 99.95% (KraMZ Ltd., Krasnoyarsk, Russia) were cut unto pieces and used as substrates. All solutions were prepared using an 80% aqueous solution of H_2_SeO_4_ (Chemcraft, Kaliningrad, Russia, analytical grade). Before anodizing, the surface of the aluminum sheets was cleaned of dust and organic contaminants by immersion in acetone in an ultrasonic bath for 10 min. The aluminum substrates were then electropolished in a perchloric acid and ethanol solution (1 part 60% HClO_2_: 6 parts 96% ethanol) at 40 V and 5 °C, followed by rinsing in deionized water. The anodizing area was 7 cm^2^ on one side of the aluminum sheet.

The prepared substrates were anodized in 0.5 M and 1.5 M selenic acid. The cell was attached to a Termex CRIO-VT-01 thermostat (Termex, Tomsk, Russia). The temperature varied between 5 and 40 °C. The electrolyte was gently stirred to ensure uniform heat distribution and prevent bubble formation and aggregation of the products on the sample surface. A platinum grid with an area of 8 cm^2^ was used as a counter electrode.

The anodization was performed in galvanostatic mode at current densities of 5 mA/cm^2^ and 15 mA/cm^2^. This mode was selected for precise control over the total charge transferred through the system. In accordance with Faraday’s law, the quantity of aluminum participating in the electrochemical reaction—and hence the thickness of the anodic aluminum oxide films—is directly proportional to the charge passed [18]. To ensure comparable film thicknesses across samples prepared at different temperatures, the current density and anodization duration were maintained constant within each experimental series. This strategy allowed for meaningful comparisons using characterization techniques sensitive to thickness. Furthermore, the galvanostatic regime facilitates the prediction of the anodization time required to produce films with sufficient mass for electron paramagnetic resonance measurements.

The selected anodization regimes were designed to encompass a range of oxide-formation conditions conducive to producing both ordered and low-ordered structures [7,8,12], while explicitly excluding hard anodization processes [8,9,12,13] and the processes causing “burning” [30]. Hard anodization conditions were avoided because the dependencies of sample composition and morphology on process parameters differ significantly from those observed under mild anodization [31], warranting separate study. “Burning” was excluded as it leads to a highly non-uniform surface morphology, characterized by localized hills and cracks alongside areas of significantly reduced oxide thickness, which complicates sample preparation and characterization and can introduce inaccuracies.

Previous research [8] has shown that in selenic acid at concentrations below 0.3 M, a current density of 5 mA/cm^2^ leads to the formation of “burning spots” (a stain-like form of “burning” [32]), while an ordered porous structure is achieved at 0.5 M. With increasing current density, the boundary between the regimes of “burning spot” formation and ordered structure development shifts toward higher electrolyte concentrations [9]. Based on these established trends, the following anodization parameters were chosen: 0.5 M H_2_SeO_4_ at 5 mA/cm^2^ and 1.5 M H_2_SeO_4_ at 15 mA/cm^2^.

Porous anodic alumina samples were fabricated using a two-step anodization procedure, a method established for producing anodic aluminum oxide with a well-ordered pore structure [33,34]. The first anodization step was conducted for 30 min. Subsequently, the sacrificial oxide layer formed during this step was removed by immersing the samples in a preheated (60 °C) solution containing 0.3 M CrO_3_ and 0.1 M H_3_PO_4_. The second anodization was then performed. For characterization by electron microscopy, X-ray diffraction, X-ray photoelectron spectroscopy, elemental analysis, and photoluminescence, this second step lasted 30 min, with the AAO layer left on the aluminum substrate. For electron paramagnetic resonance studies, the second anodization was extended to 180 min to obtain a sufficiently thick and massive oxide film, which was subsequently separated from the substrate. Voltage–time (V–t) curves for the second stage of anodization in selenic acid, carried out for 30 min at different temperatures, are presented in the Supplementary Materials (Figure S1).

Thicker AAO layers were specifically prepared for EPR measurements to ensure the sample mass was sufficient for a high signal-to-noise ratio (see Section 2.2) and to enable the oxide films to be cleanly separated from the aluminum substrate for placement into the measurement cell. Conversely, for other characterization techniques, such excessive film thickness was either unnecessary or would have introduced complications, such as significant charge accumulation in the dielectric samples, which could interfere with the measurements.

For EPR measurements, it was necessary to detach the AAO layer from the aluminum substrate because the electromagnetic field used in the technique induces conduction currents in the metallic substrate, severely degrading the quality factor (Q factor) of the measuring resonator. To achieve this separation, the aluminum oxide sample on its substrate was placed in a polytetrafluoroethylene (PTFE) cell with the aluminum side facing upward. A defined area of 6 cm^2^ was then selectively etched using a 0.7 M CuCl_2_ solution until the underlying aluminum was completely dissolved. The deposited copper layer was carefully removed with a PTFE rod, after which the sample was thoroughly rinsed with deionized water and air-dried. Finally, the resulting free-standing AAO membrane was broken into appropriately sized fragments using a sharp-tipped quartz rod for placement into a quartz ampoule.

For the purpose of comparing luminescent properties and defect characteristics, AAO samples were also synthesized in oxalic acid under analogous conditions: a current density of 15 mA/cm^2^, a temperature of 5 °C, and a duration of 30 min. However, given the limited solubility of oxalic acid in water—approximately 0.5 M at 5 °C [16]—the anodization was conducted using a 0.45 M oxalic acid solution, prepared using oxalic acid, analytical grade (Rushim, Moscow, Russia) .

### 2.2. Characterization

The morphology of the samples was characterized using a Carl Zeiss SUPRA 40 field-emission scanning electron microscope (FE-SEM) (Carl Zeiss, Oberkochen, Germany), operating with an in-lens secondary electron detector and an aperture of 30 μm. Geometric parameters of the samples were analyzed using ImageJ software, version 1.54p (National Institutes of Health, Bethesda, MD, USA) . For most samples, pore and cell diameters were determined automatically from top-view SEM images by applying a grayscale threshold. An exception was the sample anodized in 1.5 M H_2_SeO_4_ at 15 mA/cm^2^ and 40 °C, for which reliable automated measurement from the top-view image was not feasible. For this specific sample, the diameters were instead estimated from cross-sectional SEM images through multiple manual measurements taken at various locations across the sample.

XPS analysis was performed using a PHI 5000 Versa Probe II system (Physical Electronics (PHI), Chanhassen, MI, USA) equipped with a GCIB source. Prior to analysis, the sample surface was etched using monoatomic Ar^+^ ions at 2 keV, rastered over a 2 × 2 mm^2^ area for 10 min. The X-ray source was a monochromated Al Kα line (hν = 1486.6 eV, 50 W) with a 200 µm spot diameter, and a dual-charge neutralization system was employed. The chemical states of the elements were determined from high-resolution (HR) spectra. HR spectra for the O 1s, Al 2p, and Se 3d core levels were recorded with an analyzer pass energy of 23.5 eV and a step size of 0.2 eV. The binding energy (E_b_) scale was calibrated using the Au 4f peak at 83.96 eV and the Cu2p3 peak at 932.62 eV, and was subsequently corrected by referencing the Al 2p peak in Al_2_O_3_ at 74.2 eV.

Elemental analysis was conducted using a ZSX Rigaku Primus IV (Rigaku Corporation, Tokyo, Japan) wavelength-dispersive X-ray fluorescence (WDXRF) spectrometer. Selenium content was quantified using a scintillation detector with a LiF200 diffraction crystal, operating at 50 kV and 60 mA, and a 10 mm diaphragm aperture.

X-ray diffraction (XRD) analysis was performed using a Rigaku MiniFlex 600 diffractometer (Rigaku Corporation, Tokyo, Japan) with a Cu Kα radiation source (λ = 1.54 Å). Data were collected in a 2θ range from 5° to 90° with a step size of 0.01°.

Photoluminescence (PL) spectra were acquired using a PerkinElmer FL 8500 fluorescence spectrometer (PerkinElmer, Shelton, CT, USA). Measurements covered a spectral range of 250 to 900 nm, enabling characterization of emission across the ultraviolet, visible, and near-infrared regions, with a spectral slit width of 1 nm. Following preliminary evaluation, an excitation wavelength of 360 nm was selected for the final measurements.

Electron paramagnetic resonance measurements were conducted using an ELEXSYS-E500-10/12 spectrometer (Bruker, Karlsrue, Germany), operating at an X-band frequency of 9.5 GHz with a sensitivity of 5 × 10^10^ spins/G. The samples were loaded into 4 mm diameter quartz ampoules for analysis.

Of note, the EPR technique is based on the absorption of microwave radiation by states containing unpaired electrons, known as paramagnetic centers, within a sample exposed to an external static magnetic field. This field removes the degeneracy in the magnetic quantum number, thereby splitting the energy levels. The transitions between these levels are recorded as the EPR signal. The design of EPR spectrometers allows the microwave absorption signal from paramagnetic centers to be collected from the entire sample volume. As a result, a larger sample mass within the measuring cell yields a more intense EPR signal and a superior signal-to-noise ratio. For this reason, thick porous aluminum oxide layers were specifically fabricated for the EPR experiments.

The g-factors were calculated using the fundamental electron paramagnetic resonance condition: g = hν/μ_B_H, where h is Planck’s constant, ν is the microwave frequency, μ_B_ is the Bohr magneton, and H is the external magnetic field strength at resonance. The concentration of paramagnetic centers was quantified relative to a CuCl_2_·2H_2_O standard with a known spin concentration. This involved a double integration of the first-derivative EPR signals: first, to obtain the absorption line, and second, to determine the area under this curve for both the sample and the standard. The concentration was then derived using the formulas: N^abs^_AAO_ = N_st_ · (S_AAO_/S_st_) and N_s_ = N^abs^_AAO_/m, where N^abs^_AAO_ is the absolute number of spins in the AAO sample, N_st_ is the known number of spins in the standard (6 × 10^18^), S_AAO_ and S_st_ are the integrated signal areas for the sample and standard, respectively, N_s_ is the spin concentration per gram, and m is the mass of the AAO sample in grams. Critical to this comparative method, the spectra for all samples and the standard were recorded under identical instrumental parameters (microwave power, modulation amplitude, time constant, temperature, number of scans, and receiver gain). The calculations were performed using a custom-written program.

## 3. Results and Discussion

### 3.1. Morphology

**Figure 1:**
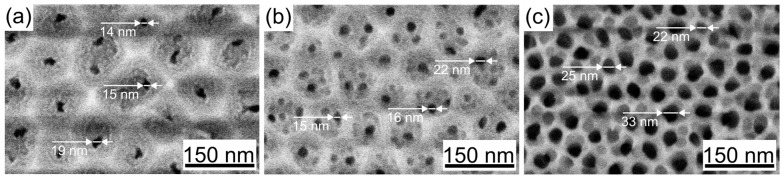
SEM images of porous anodic alumina formed in 0.5 M H_2_SeO_4_ at a current density of 5 mA/cm^2^ at temperatures: (**a**) 5 °C; (**b**) 25 °C; (**c**) 40 °C.

The influence of anodizing temperature on the geometric parameters of porous AAO formed in a H_2_SeO_4_ electrolyte was analyzed. Surface images of AAO produced at various temperatures are presented in Figure 1.

The sample anodized at 5 mA/cm^2^ in 0.5 M H_2_SeO_4_ at 5 °C exhibits a porous layer with hexagonal ordering, approaching a structure where each cell contains a single large pore (Figure 1a). Furthermore, the cells clearly consist of two distinct layers: a brighter inner layer and a darker outer layer. This morphology corresponds to the descriptions of double-layer porous cells reported in [35,36,37], where the inner layer is characterized as a compact, low-defect structure, while the outer layer has a spongy morphology containing embedded electrolyte ions.

The porous layer formed at 5 mA/cm^2^ in 0.5 M H_2_SeO_4_ at 5 °C displays a hexagonal arrangement, closely approximating a structure where each cell contains a single large pore (Figure 1a). Additionally, the cells distinctly reveal a two-layer composition: a brighter inner layer and a darker outer layer. This observed structure aligns with prior descriptions of double-layer porous cells [35,36,37], in which the inner layer is noted for its compact, low-defect morphology, whereas the outer layer is spongy and incorporates embedded electrolyte ions.

The increase in anodization temperature to 25 °C leads to a disordering of the porous structure, characterized by the formation of multiple small-diameter pores within a single cell (Figure 1b). Concurrently, the porous cell diameter decreases—a trend analogous to that observed with reduced current density [2]. The double-layer structure of the cells, however, remains evident. Upon further temperature increase, the cell diameter continues to diminish while the pore diameter expands, a result of the enhanced chemical etching rate of the pore walls. This process leads to a significant reduction in the thickness of the outer layer (Figure 1c).

**Figure 2:**
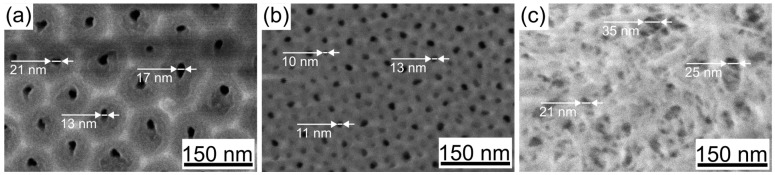
SEM images of porous anodic alumina formed in 1.5 M H_2_SeO_4_ at a current density of 15 mA/cm^2^ at temperatures: (**a**) 5 °C; (**b**) 25 °C; (**c**) 40 °C.

**Figure 3:**
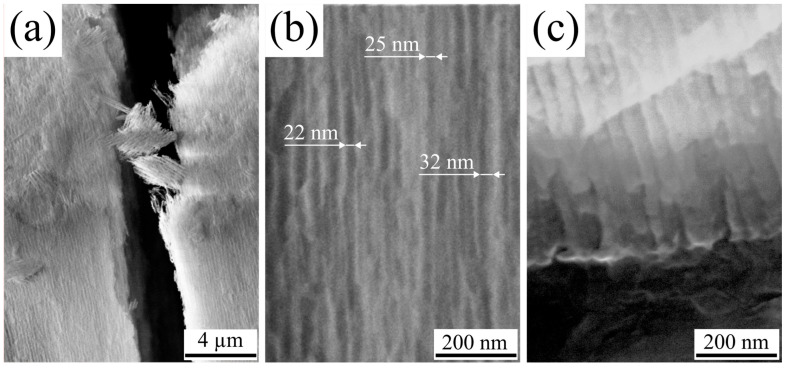
Side view of a porous anodic alumina formed in 1.5 M H_2_SeO_4_ at a current density of 15 mA/cm^2^ at 40 °C: (**a**) the upper part; (**b**) the central part; (**c**) the inner part of the oxide.

These trends become more pronounced with increasing anodization temperature in more concentrated electrolytes, such as 1.5 M H_2_SeO_4_ (Figure 2). It is important to note that the higher electrolyte concentration necessitated a correspondingly higher applied current density. This is because an increased current density elevates the oxide growth rate and helps maintain sufficient mechanical stress within the films, which are factors known to promote the formation of an ordered porous structure [9,30]. Consequently, ordered nanostructures were successfully formed under the following conditions: 15 mA/cm^2^ in 1.5 M H_2_SeO_4_ at 5 °C (Figure 2a). Raising the temperature to 25 °C resulted in structural disordering and the formation of numerous small-diameter pores (Figure 2b), accompanied by a significant reduction in cell diameter (from 84 ± 7 nm to 52 ± 5 nm). A further temperature increase (to 40 °C) intensified etching of the pore walls, leading to severe wall thinning and fragmentation of the upper part of porous layer (Figure 2c and Figure 3a), which rendered its top surface unmeasurable. Despite this, the inner and central regions of the sample retained a cylindrical pore structure (Figure 3b,c), featuring larger pore diameters and smaller cell diameters compared to samples formed at 5 °C and 25 °C.

The results of the morphological characterization for AAO samples fabricated under different processing conditions in H_2_SeO_4_ solutions are summarized in Table 1.

Based on scanning electron microscopy analysis, the porosity of oxide matrices formed in 0.5 M H_2_SeO_4_ was found to range from 1.5% for samples prepared at 5 °C to 16.5% for those prepared at 40 °C. Increasing the electrolyte concentration to 1.5 M drastically intensifies the chemical etching of the pore walls, causing porosity to rise from 1.7% at low temperatures to unmeasurable values at higher temperatures. In the latter case, the pore wall thickness in the upper part of the oxide decreased significantly, resulting in the oxide flaking off and disintegrating into individual fibers.

Given that the trends observed with increasing temperature were consistent for both 0.5 M and 1.5 M electrolyte concentrations—yet more pronounced in the 1.5 M samples—only the results for AAO formed in 1.5 M H_2_SeO_4_ are presented in the following sections.

### 3.2. Chemical and Crystal Structure

**Figure 4:**
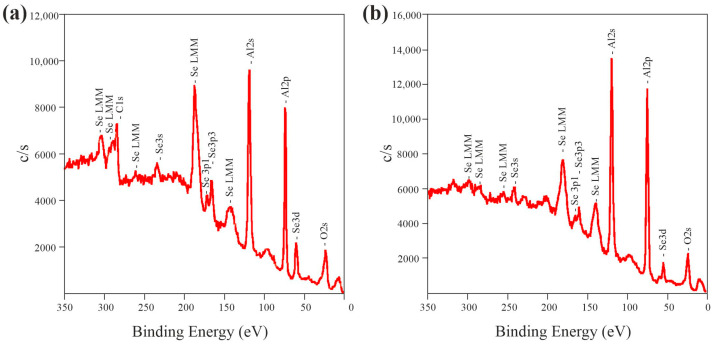
XPS spectra of porous anodic alumina formed in 1.5 M H_2_SeO_4_ at a current density of 15 mA/cm^2^ at 5 °C: (**a**) before and (**b**) after ion-beam etching.

The elemental composition and chemical bonds were investigated using X-ray photoelectron spectroscopy (XPS). Representative XPS spectra of AAO formed in a selenic acid solution are presented in Figure 4.

The spectra of the samples formed at different temperatures do not vary significantly, although the intensity of the peaks related to Se slightly decreases with increasing anodization temperature. Atomic concentrations were determined by the method of relative elemental sensitivity factors from the survey spectrum, using the integrated intensities of the following lines: C 1s, O 1s, Al 2p and Se 3d. These results are shown in Table 2.

The XPS data indicate a higher selenium concentration on the sample surfaces. This suggests that selenium is partially present due to its adsorption from the electrolyte onto the AAO surface, and its incomplete removal during the washing step. It is worth noting that selenium may exist in both selenate and elemental forms, as the anodic oxidation process can induce the partial disproportionation of selenic acid into selenide anions and even Se^0^ species [7,21]. The selenium content decreases from approximately 2 at.% to less than 1 at.% as the anodization temperature increases.

**Figure 5:**
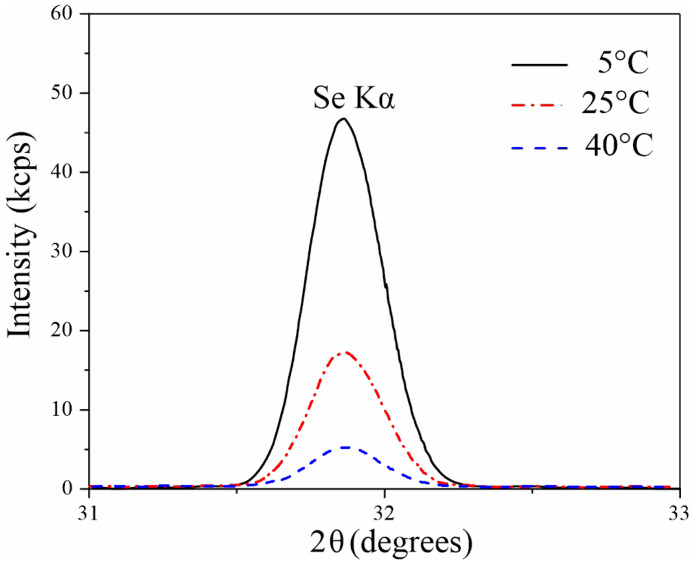
WDXRF spectra of anodic alumina prepared in 1.5 M H_2_SeO_4_ at a current density of 15 mA/cm^2^ at different anodization temperatures.

Given that the measured selenium concentrations approached the detection limit, particularly for the sample formed at 40 °C, complementary wavelength-dispersive X-ray fluorescence (WDXRF) analysis was conducted to better quantify the selenium content in films synthesized at different temperatures (Figure 5). The WDXRF spectra showed a distinct selenium peak at 2θ = 31.87 degrees. These results are consistent with the XPS data, showing a decrease in selenium signal intensity with increasing electrolyte temperature. Specifically, the intensity of the selenium peak in samples formed at 5 °C was 2.6 and 7.5 times greater than in AAO formed at 25 °C and 40 °C, respectively.

To the best of our knowledge, comparative studies of AAO composition as a function of synthesis temperature have not been previously reported. Nevertheless, our results for the sample fabricated at 5 °C can be compared with data for similar systems. The results of our XPS and WDXRF analyses (1–2%) are consistent with the values obtained by Kamnev et al. for Al_2_O_3_/ZrO_2_ and Al_2_O_3_/NbO_2_ composites, although these objects are different: the composites are formed by the interaction of growing ZrO_2_ or NbO_2_ nanocolumns with the narrow outer part of the AAO, which is more contaminated with Se [11,21]. Furthermore, an overall Se content of 1.6 at.% (measured by EDX) was reported for AAO formed in 0.3 M H_2_SeO_4_ at 5 °C, which correlates with our data, though that study did not account for the depth-dependent concentration gradient of Se observed in our work. In contrast, EDX analysis in [20] reported significantly higher Se concentrations (9 to 25 at.%) for AAO formed at 0 °C under varying anodization voltages. This discrepancy is likely attributable to the lower synthesis temperature and the use of hard anodization regimes in that study, a trend consistent with our observation of higher Se incorporation at lower temperatures. Additionally, correct quantification via EDX is complicated by the overlap of Al and Se X-ray emission lines (K and L lines) within the same energy range (~1.4 keV) [38].

**Figure 6:**
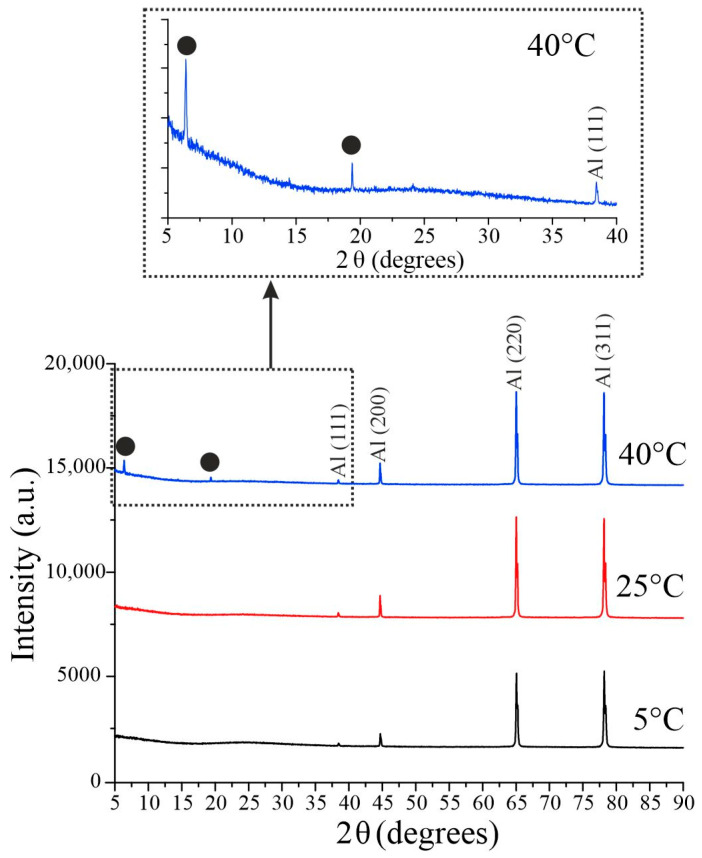
Diffraction patterns of samples prepared in 1.5 M H_2_SeO_4_ at a current density of 15 mA/cm^2^ at different anodization temperatures. Circles denote the small-angle reflections, which can be indexed to γ-Al_2_O_3_.

Figure 6 presents the X-ray diffraction patterns of samples prepared at different electrolyte temperatures. For anodization temperatures of 5 °C and 25 °C, all detected peaks correspond to the aluminum substrate (JCPDS Card No. 04-0787), confirming that the porous AAO matrix itself is amorphous. Notably, when the temperature is increased to 40 °C, two new peaks emerge at 6.41° and 19.36°, which can be indexed to γ-Al_2_O_3_ (JCPDS Card No. 29-0063). This suggests the onset of crystallization within the aluminum oxide film. This effect was reproducible across both similar substrates and substrates of differing thicknesses and grades.

It has previously been reported that porous aluminum oxide, which typically consists of an anion-free amorphous layer, can undergo crystallization when irradiated by an electron beam, such as during examination in a scanning electron microscope. Although the presence of crystalline alumina has not been confirmed in porous films formed in phosphoric, oxalic, or sulfuric acids, anodization in a mixture of sulfuric and chromic acid has been found to yield amorphous alumina with traces of γ-Al_2_O_3_ [39]. These structural features were observed in samples synthesized at 20 °C; therefore, the formation of a crystalline phase in samples produced in electrolytes heated to 40 °C is also plausible.

### 3.3. Luminescent Properties and Paramagnetic Centers

**Figure 7:**
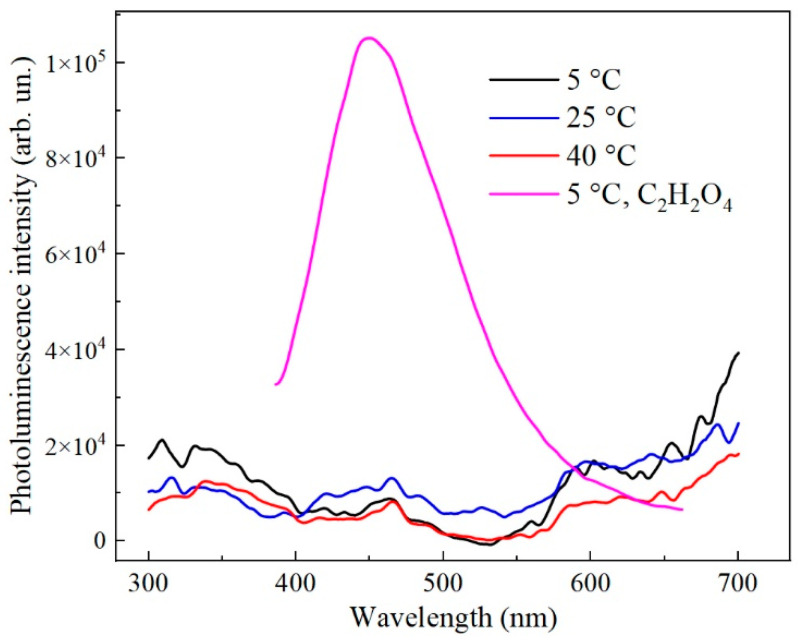
Photoluminescence spectra of porous alumina synthesized in 1.5 M H_2_SeO_4_ at 5–40 °C and 0.45 M C_2_H_2_O_4_ at 5 °C under the same current densities (15 mA/cm^2^) and anodization durations (30 min).

Photoluminescence spectra of porous AAO samples synthesized in 1.5 M H_2_SeO_4_ at temperatures ranging from 5 to 40 °C are shown in Figure 7. The samples exhibit only weak luminescence, close to the noise level, with no distinct peaks or bands observed across the entire spectral range. Variations in the anodization temperature do not produce any substantial alterations in the PL spectra.

**Figure 8:**
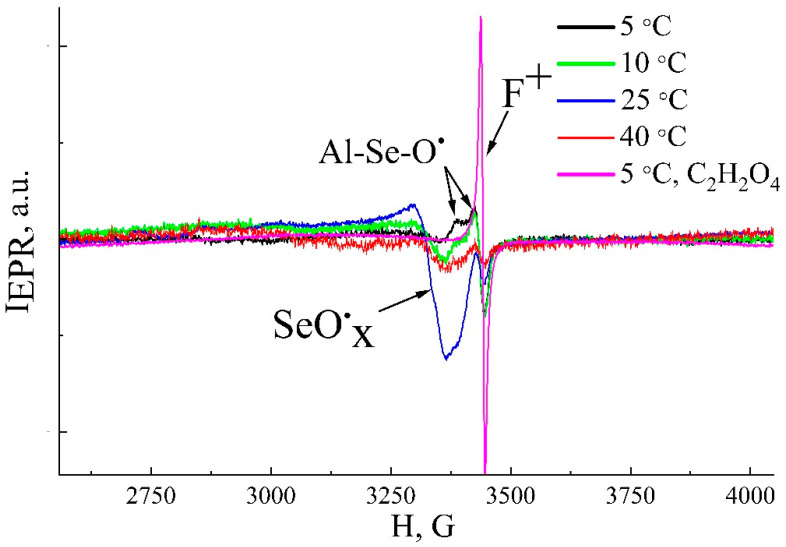
Comparative EPR spectra of porous anodic alumina samples synthesized in 1.5 M H_2_SeO_4_ at 5–40 °C and in 0.45 M C_2_H_2_O_4_ at 5 °C under the same current densities (15 mA/cm^2^) and anodization durations (180 min).

The EPR spectra of samples prepared at different temperatures are shown in Figure 8. It is evident that all spectra exhibit a complex shape resulting from the superposition of several distinct signals, indicating the presence of multiple types of paramagnetic centers. All samples display a resonance line at g_I_ = 2.0024 ± 0.0005. This signal has been frequently observed in prior EPR studies of AAO formed in various electrolytes and is attributed to oxygen vacancies with unpaired electrons, known as F^+^-centers, within the aluminum oxide matrix [27,28,29,40].

The EPR signal corresponding to F^+^ centers overlaps with an anisotropic signal characterized by g_II1_ = 2.0322 ± 0.0005 and g_II2_ = 2.0081 ± 0.0005. These g-factor values are indicative of an electron localized on an oxygen atom in a configuration similar to O^−^, a type of paramagnetic center previously observed in various metal oxides [41]. This signal is therefore attributed to an oxygen radical with one unpaired electron, where the second bond pairs it with another atom. As this EPR feature is unique to samples formed in selenic acid and absent in AAO from other electrolytes, the oxygen is likely bonded to a selenium atom (>Al-Se-O*). Furthermore, the signal’s persistence after extensive etching, washing, and drying—disappearing only upon near-complete dissolution of the pore walls—confirms that it originates from ions incorporated into the oxide lattice, rather than from residual electrolyte. Since this radical is not observed in AAO formed in structurally similar sulfuric acid (which does not decompose to sulfides), it is hypothesized to be a decomposition product of selenic acid (e.g., to selenides). A third signal with g_III_ = 2.0735 ± 0.0005 is also identified. This signal appears in samples anodized at temperatures ≥ 10 °C, reaching maximum intensity at 25 °C. It resembles signals in sulfuric-acid-formed oxides associated with sulfate complexes [15,24] and, given the structural similarity of sulfuric and selenic acids as well as comparable g-factors reported for SeO_4_^−^ radicals in Na_2_SeO_4_ [42], it is assigned to a selenate-like radical containing an oxygen atom with an unpaired electron.

The intensity of the F^+^ center signal decreases with rising anodization temperature. The maximum concentration of F^+^ centers, N_sF_ = 8.2 × 10^15^ g^−1^, is found in AAO formed at 5 °C. The concentration of the >Al-Se-O* centers (g_II_ ≈ 2.0322) is highest (N_sO_ ≈ 10^17^ g^−1^) at low temperatures (5–10 °C) and diminishes with increasing temperature. In contrast, the intensity of the selenate-like radical signal (g_III_ ≈ 2.0735) increases up to 25 °C, where its concentration rises sharply to N_sS_ = 6 × 10^18^ g^−1^. For samples synthesized at 40 °C, the intensities of all EPR signals, and thus the defect concentrations, decrease significantly (Figure 8).

Given that the PL of the samples is extremely weak and shows no substantial variation with synthesis temperature between 5 and 40 °C (Figure 7), it can be concluded that the oxygen-related radicals (defects with g_II_ and g_III_) are not the source of PL. Literature reports consistently attribute PL in porous alumina to oxygen vacancies (F^+^ centers) [27,28,29,40]. In our samples, the concentration of F^+^ centers is relatively low (not exceeding ~10^16^ g^−1^), which explains the weak PL observed. To verify this hypothesis, AAO samples were synthesized in oxalic acid for comparison, as such structures are known to exhibit intense PL [27,28,40]. Indeed, these oxalic-acid-formed AAO samples show strong PL (Figure 7). EPR analysis of these samples confirms the presence of oxygen vacancies (Figure 8) at a concentration (N_sF_ = 2 × 10^17^ g^−1^) an order of magnitude higher than in the selenic acid samples. Notably, increasing the concentration of oxygen-containing radicals to 6 × 10^18^ g^−1^ in the H_2_SeO_4_-formed samples does not enhance the PL. Therefore, the PL intensity correlates directly with the concentration of F^+^ centers and can be modulated by the choice of electrolyte.

**Figure 9:**
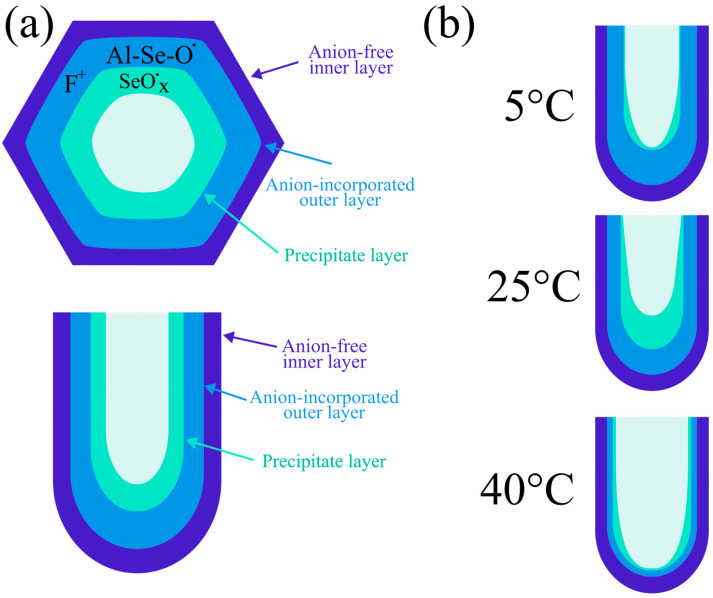
Schematic representation of the cell structure of porous alumina formed in a selenic acid electrolyte, showing different paramagnetic centers location for samples (**a**) and changes in the aluminum oxide structure at different temperatures (**b**).

To summarize the behavior of paramagnetic centers regarding anodization temperature, a schematic model of the AAO cell structure illustrating the different paramagnetic centers and their proposed locations is provided in Figure 9.

As noted in Section 3.1 (“Morphology”), increasing the synthesis temperature enhances the etching of the oxide walls. This reduces the relative proportion of the anion-incorporated outer layer within the total AAO volume. Consequently, the concentration of oxide defects corresponding to the g_I_ and g_II_ EPR signals gradually decreases—both as the synthesis temperature rises from 5 °C to 40 °C and when the outer layer is thinned by additional post-synthesis etching. This behavior indicates that the defects responsible for these signals are located and uniformly distributed within the outer layer of the oxide.

Interpreting the g_III_ EPR signal is more complex. Its intensity increases with rising synthesis temperature, but only up to 25 °C. Compositional analysis by XPS and WDXRF does not show a significant increase in selenium concentration or a change in its valence state compared to the sample synthesized at 5 °C. However, the samples analyzed by these techniques are considerably thinner. It is possible that the g_III_ signal arises from precipitates within a thin surface layer of the AAO (Figure 9b). At lower temperatures, the electrolyte’s chemical activity is reduced and the pores are wider, potentially resulting in fewer adsorbed particles on the oxide walls. As the temperature increases, the electrolyte becomes more active and the pores narrow, which may promote the precipitation of reactive species. In the thinner samples used for XPS and WDXRF, the oxide is rinsed more effectively than in the much thicker samples prepared for EPR. This could explain why the surface-sensitive techniques do not detect compositional changes that would account for the emergence and growth of the g_III_ signal. In contrast, in the thicker EPR samples, the precipitates are retained and detected, highlighting the superior sensitivity of EPR for tracing such compositional variations.

The decrease in concentration for all defect types at 40 °C can be attributed to the extensive etching of the porous walls, which removes the defective layer containing incorporated ions, other species, and vacancies. This leaves behind a more perfect, nearly crystalline aluminum oxide layer, consistent with the XRD results. Consequently, the total defect concentration in this layer approaches zero (Figure 9b).

## 4. Conclusions

Based on a comprehensive study of AAO films formed in selenic acid at different anodizing temperatures, a number of important conclusions can be drawn from both fundamental and practical points of view. It has been established that with an increase in the synthesis temperature, the diameter of the AAO cells decreases. Meanwhile, the etching of AAO walls increases, causing an increase in pore diameter and even leading to the disintegration of AAO formed in 1.5 M H_2_SeO_4_ into individual fibers at 40 °C. The concentration of selenium in the samples does not exceed 2 at.%. When the temperature increases to 40 °C, two peaks appear in the XRD pattern at 6.41° and 19.36°, which may indicate the beginning of crystallization of the aluminum oxide. The samples exhibit extremely weak luminescence without pronounced PL bands. In AAO formed in a selenic acid electrolyte, various paramagnetic centers were discovered: F^+^ centers; for the first time, centers in which the electron belongs to an oxygen atom in a configuration similar to O^−^ (>Al-Se-O*); and paramagnetic defects structurally similar to selenates (g_III_ = 2.0735 ± 0.0005). Using test experiments with AAO samples synthesized in oxalic acid, it was established that, of all the defects found in AAO synthesized in selenic acid, only F^+^ centers are responsible for luminescence. With the increase in the electrolyte temperature, the selenium content, luminescence intensity, and concentration of F^+^ centers and (>Al-Se-O*)-centers decrease. The concentration of all types of centers decreases drastically at 40 °C. These trends can be explained by the significant etching of the porous structure walls, which removes the defective outer layer containing both embedded impurities from the electrolyte and oxygen vacancies. As a result, only the inner alumina layer, with an anion-free, near-crystalline structure, remains, which is confirmed by XRD results. A geometric model is proposed that explains the results obtained for the set of AAO samples synthesized at different temperatures. The obtained results are new and original and can be purposefully used for the manufacturing of AAO with specified optical properties.

## Figures and Tables

**Table 1:** Main morphological parameters of the porous anodic alumina synthesized in H_2_SeO_4_ at different temperatures.

Sample	Pore Diameter, nm	Cell Diameter, nm	Porosity (SEM + Fiji), %
0.5 M-5 °C-5 mA/cm^2^	15 ± 6	114 ± 10	1.5
0.5 M-25 °C-5 mA/cm^2^	16 ± 9	101 ± 8	7.5
0.5 M-40 °C-5 mA/cm^2^	25 ± 6	58 ± 6	16.5
1.5 M-5 °C-15 mA/cm^2^	17 ± 4	84 ± 7	2.0
1.5 M-25 °C-15 mA/cm^2^	11 ± 4	52 ± 5	4.2
1.5 M-40 °C-15 mA/cm^2^ *	25 ± 10	37 ± 10	45.5

* The pore and cell diameter values for the sample (1.5 M, 40 °C, 15 mA/cm^2^) are approximate estimates derived from cross-sectional images of the AAO’s bottom region. The upper part could not be measured due to the etching of the alumina into individual fibers.

**Table 2:** Chemical content (at.%) of porous anodic alumina formed in 1.5 M H_2_SeO_4_ before and after ion-beam etching.

Sample	Ion-Beam Etching	C	O	Al	Se	Se/Al
1.5 M-5 °C-15 mA/cm^2^	-	10.2	60.4	27.4	2.0	0.07
	10 min	-	60.4	38.2	1.4	0.04
1.5 M-25 °C-15 mA/cm^2^	-	5.1	62.3	30.2	1.8	0.06
	10 min	-	59.7	39.1	1.2	0.03

## Data Availability

The raw data supporting the conclusions of this article will be made available by the authors on request.

## Supplementary Materials

The following supporting information can be downloaded at: https://www.mdpi.com/article/10.3390/nano15241855/s1, Figure S1: Voltage–time (V–t) curves for the second stage of anodization in selenic acid at different temperatures. AAO synthesized in: (a) 0.5 M H_2_SeO_4_ at a current density of 5 mA/cm^2^, (b) 1.5 M H_2_SeO_4_ at a current density of 15 mA/cm^2^.

## References

[1] Santos A., Kumeria T., Losic D. (2013). Nanoporous anodic aluminum oxide for chemical sensing and biosensors. Trends Anal. Chem..

[2] Abd-Elnaiem A.M., Mohamed Z.A., Soliman S.E., Almokhtar M. (2024). Synthesis, characterization, and optical sensing of hydrophilic anodic alumina films. Opt. Mater..

[3] Sadykov A.I., Kushnir S.E., Roslyakov I.V., Baranchikov A.E., Napolskii K.S. (2019). Selenic Acid Anodizing of Aluminium for Preparation of 1D Photonic Crystals. Electrochem. Commun..

[4] Zhao X., Meng G., Han F., Li X., Chen B., Xu Q., Zhu X., Chu Z., Kong M., Huang Q. (2013). Nanocontainers made of various materials with tunable shape and size. Sci. Rep..

[5] Sacco L.N., Vollebregt S. (2023). Overview of Engineering Carbon Nanomaterials Such As Carbon Nanotubes (CNTs), Carbon Nanofibers (CNFs), Graphene and Nanodiamonds and Other Carbon Allotropes inside Porous Anodic Alumina (PAA). Nanomaterials.

[6] Brzózka A., Brudzisz A., Hnida K., Sulka G.D., Losic D., Santos A. (2015). Chemical and Structural Modifications of Nanoporous Alumina and Its Optical Properties. Electrochemically Engineered Nanoporous Materials.

[7] Nishinaga O., Kikuchi T., Natsui S., Suzuki R.O. (2013). Rapid fabrication of self-ordered porous alumina with 10-/sub-10-nm-scale nanostructures by selenic acid anodizing. Sci. Rep..

[8] Nazarkina Y., Gavrilov S., Terryn H., Petrova M., Ustarroz J. (2015). Investigation of the Ordering of Porous Anodic Alumina Formed by Anodization of Aluminum in Selenic Acid. J. Electrochem. Soc..

[9] Nazarkina Y., Kamnev K., Dronov A., Dudin A., Pavlov A., Gavrilov S. (2017). Features of Porous Anodic Alumina Growth in Galvanostatic Regime in Selenic Acid Based Electrolyte. Electrochim. Acta.

[10] Ahmadzadeh M., Kashi M.A., Noormohammadi M., Ramazani A. (2021). Small-diameter magnetic and metallic nanowire arrays grown in anodic porous alumina templates anodized in selenic acid. Appl. Phys. A Mater. Sci. Process..

[11] Kamnev K., Bendova M., Fohlerova Z., Fialova T., Martyniuk O., Prasek J., Cihalova K., Mozalev A. (2025). Arrays of ultra-thin selenium-doped zirconium-anodic-oxide nanorods as potential antibacterial coatings. Mater. Chem. Front..

[12] Gordeeva E.O., Roslyakov I.V., Napolskii K.S. (2019). Aluminium anodizing in selenic acid: Electrochemical behaviour, porous structure, and ordering regimes. Electrochim. Acta.

[13] Akiya S., Kikuchi T., Natsui S., Suzuki R.O. (2015). Optimum Exploration for the Self-Ordering of Anodic Porous Alumina Formed via Selenic Acid Anodizing. J. Electrochem. Soc..

[14] Wood G., O’Sullivan J.P. (1970). The anodizing of aluminium in sulphate solutions. Electrochim. Acta.

[15] Kytina E.V., Konstantinova E.A., Pavlikov A.V., Nazarkina Y.V., Dronova D.A. (2025). Influence of Synthesis Conditions on the Photoluminescence Properties and Paramagnetic Centers of Porous Alumina Formed in Sulfuric Acid. Bull. Russ. Acad. Sci. Phys..

[16] Cheng C., Ng K.Y., Ngan A.H.W. (2011). Quantitative characterization of acid concentration and temperature dependent self-ordering conditions of anodic porous alumina. AIP Adv..

[17] Stępniowski W.J., Bojar Z. (2011). Synthesis of anodic aluminum oxide (AAO) at relatively high temperatures. Study of the influence of anodization conditions on the alumina structural features. Surf. Coat. Technol..

[18] Zaidi S.M.J., Butt M.Z. (2018). First-Step Anodization of Commercial Aluminum in Oxalic Acid: Activation Energy of Rate Process and Structural Features of Porous Alumina and of Aluminum Substrate as a Function of Temperature. J. Chem. Soc. Pak.

[19] Leontiev A.P., Roslyakov I.V., Napolskii K.S. (2019). Complex Influence of Temperature on Oxalic Acid Anodizing of Aluminium. Electrochim. Acta.

[20] Sadykov A.I., Leontev A.P., Kushnir S.E., Napolskii K.S. (2021). Kinetics of the Formation and Dissolution of Anodic Aluminum Oxide in Electrolytes Based on Sulfuric and Selenic Acids. Russ. J. Inorg. Chem..

[21] Kamnev K., Bendova M., Pytlicek Z., Prasek J., Kejík L., Güell F., Llobet E., Mozalev A. (2023). Se-doped Nb_2_O_5_–Al_2_O_3_ composite-ceramic nanoarrays via the anodizing of Al/Nb bilayer in selenic acid. Ceram. Int..

[22] Green S., Badan J.A., Gilles M., Cortes A., Riveros G., Ramirez D., Gomez H., Quagliata E., Dalchiele E.A., Marotti R.E. (2007). Optical properties of nanoporous Al_2_O_3_ obtained by aluminum anodization. Phys. Status Solidi (c).

[23] Białek E., Włodarski M., Norek M. (2020). Fabrication of porous anodic alumina (PAA) by high-temperature pulse-anodization: Tuning the optical characteristics of PAA-based DBR in the NIR-MIR region. Materials.

[24] Kytina E.V., Konstantinova E.A., Pavlikov A.V., Nazarkina Y.V. (2025). Effect of Synthesis Parameters on Photoluminescence of Dyes in Pores of Anodic Aluminum Oxide. Russ. J. Phys. Chem. B.

[25] Nazarkina Y., Kamnev K., Polokhin A. (2017). The Effect of Annealing on the Raman Spectra of Porous Anodic Alumina Films Formed in Different Electrolytes. Proceedings of the 2017 IEEE Russia Section Young Researchers in Electrical and Electronic Engineering Conference, ElConRus 2017.

[26] Nazarkina Y., Gavrilov S.A., Polohin A.A., Gromov D., Shaman Y.P. (2016). Application of porous alumina formed in selenic acid solution for nanostructures investigation via Raman spectroscopy. Proc. SPIE Int. Soc. Opt. Eng..

[27] Huang G.S., Wu X.L., Mei Y.F., Shao X.F., Siu G.G. (2003). Strong blue emission from anodic alumina membranes with ordered nanopore array. J. Appl. Phys..

[28] Chen J.H., Huang C.P., Chao C.G., Chen T.M. (2006). The investigation of photoluminescence centers in porous alumina membranes. Appl. Phys. A.

[29] Staninski K., Kaczmarek M. (2021). Afterglow luminescence phenomena in the porous anodic alumina. Opt. Mater..

[30] Ono S., Saito M., Ishiguro M., Asoh H. (2004). Controlling Factor of Self-Ordering of Anodic Porous Alumina. J. Electrochem. Soc..

[31] Schwirn K., Lee W., Hillebrand R., Steinhart M., Nielsch K., Gösele U. (2008). Self-Ordered Anodic Aluminum Oxide Formed by H_2_SO_4_ Hard Anodization. ACS Nano.

[32] Patermarakis G. (2014). Thorough electrochemical kinetic and energy balance models clarifying the mechanisms of normal and abnormal growth of porous anodic alumina films. J. Electroanal. Chem..

[33] Masuda H., Hasegwa F., Ono S. (1997). Self-Ordering of Cell Arrangement of Anodic Porous Alumina Formed in Sulfuric Acid Solution. J. Electrochem. Soc..

[34] Stępniowski W.J., Zasada D., Bojar Z. (2011). First step of anodization influences the final nanopore arrangement in anodized alumina. Surf. Coat. Technol..

[35] Thompson G.E., Furneaux R.C., Wood G.C. (1978). Nucleation and growth of porous anodic films on Aluminium. Nature.

[36] Le Coz F., Arurault L., Datas L. (2010). Chemical analysis of a single basic cell of porous anodic aluminium oxide templates. Mater. Charact..

[37] Chang Y., Ling Z., Liu Y., Hu X., Li Y. (2012). A simple method for fabrication of highly ordered porous α-alumina ceramic membranes. J. Mater. Chem..

[38] MyScope Training. https://www.myscope.training.

[39] Parkhutik V.P., Belov V.T., Chernyckh M.A. (1990). Study of aluminium anodization in sulphuric and chromic acid solutions—II. Oxide morphology and structure. Electrochim. Acta.

[40] Khan G.G., Singh A.K., Mandal K. (2013). Structure dependent photoluminescence of nanoporous amorphous anodic aluminium oxide membranes: Role of F^+^ center defects. J. Lumin..

[41] Kokorin A.I., Kokorin A.I., Bahnemann D.W. (2003). Electron Spin Resonance of Nanostructured Oxide. Semiconductors: Chemical Physics of Nanostructured Semiconductors.

[42] Atkins P.W., Symons M.C.R. (1967). The Structure of Inorganic Radicals: An Application of Electron Spin Resonance to the Study of Molecular Structure.

