# Supplementary material for: Effect of Anodization Temperature on the Morphology and Structure of Porous Alumina Formed in Selenic Acid Electrolyte
Source: Nanomaterials (Basel). 2025 Dec 11;15(24):1855. doi: 10.3390/nano15241855 (PMC12735827; doi:10.3390/nano15241855)
Supplement: Supplementary file 1 [file nanomaterials-15-01855-s001.zip › nanomaterials-4015228-supplementary.pdf]

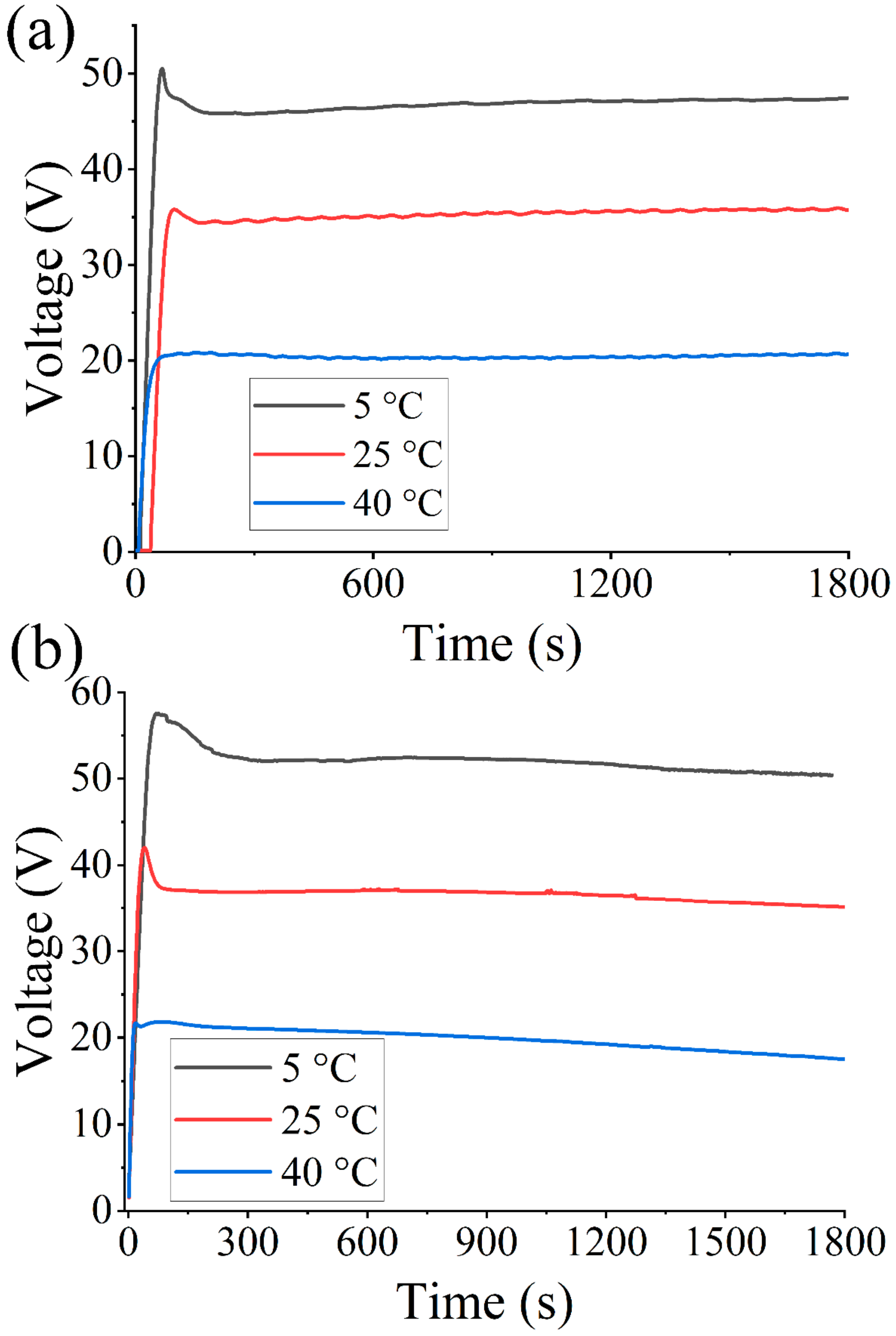

Figure S1. Voltage-time anodization curves (V-t) for anodization in selenic acid at different temperatures. AAO synthesized in: (a) 0.5 M H_2SeO_4 at a current density of 5 mA/cm^2, (b) 1.5 M H_2SeO_4 at a current density of 15 mA/cm^2
